# Glial Cell and Perineuronal Net Interactions in the Dorsal Striatum of Aged Mice

**Published:** 2026

**Authors:** Zachary A Colon, Alejandro Gamboa, Scott Litwiler, Kathleen A Maguire-Zeiss

**Affiliations:** 1 Interdisciplinary Program in Neuroscience, Georgetown University School of Medicine, Washington, DC, USA; 2 Department of Neuroscience, Georgetown University School of Medicine, Washington, DC, USA; 3 Department of Biology, Georgetown College of Arts & Sciences, Washington, DC, USA

## Commentary

Elucidating how normal aging increases vulnerability to neurodegeneration remains a major gap in our understanding of disease risk and progression. The dorsal striatum serves as the primary input nucleus of the basal ganglia and is a key region implicated in multiple neurodegenerative diseases (NDDs) [1]. In Colon *et al.* 2025 [2], we examined the impact of normal aging on neuroinflammatory signaling and perineuronal net (PNN) homeostasis within the dorsal striatum. We observed age-associated shifts in the inflammatory landscape and evidence of increased microglial activation, yet PNN homeostasis was largely preserved [2]. PNNs are highly organized extracellular matrix (ECM) specializations that preferentially enwrap the soma and proximal dendrites of fast-spiking GABAergic parvalbumin (PV) interneurons, where they contribute to the regulation of synaptic plasticity and provide protection against oxidative stress [3,4]. Building on these findings, we developed a working hypothesis to explain the apparent preservation of PNN homeostasis despite an aging-associated pro-inflammatory environment.

The shift toward a pro-inflammatory milieu, together with increased gliosis and phagocytic activity, would be expected to impact on the maintenance and integrity of perineuronal nets. The observed increase in phagocytosis-related markers may reflect microglia-directed activity as well as contributions from additional central nervous system (CNS) cell populations. Microglia are specialized embryonic-derived myeloid cells that serve as the resident immune cells of the brain and contribute to PNN homeostasis under physiological conditions [5]. In Colon *et al.* 2025, we observed evidence of microgliosis (e.g., morphological changes, *Iba1, Trem2*) along with elevated expression of markers associated with phagocytosis (e.g., *Cd68*) and extracellular matrix–modifying proteases (e.g., *Mmp9, Adam17*) capable of cleaving key PNN components [2]. Importantly, *Cd68* expression is not exclusive to microglia and has been detected in brain infiltrating macrophages, reactive astrocytes, and neutrophils during inflammation [6– 8]. Thus, increased *Cd68* levels may not solely reflect microglial phagocytic activation but may also reflect astrocyte reactivity and phagocytic phenotypes. Furthermore, astrocytes are the most abundant glial cell in the brain, and they play a major role in maintaining CNS homeostasis by regulating extracellular neurotransmitter concentrations, providing metabolic support, contributing to the synthesis and remodeling of PNN components, and modulating neuronal communication through their involvement in the tetrapartite synapse [9–12]. Astrocytes can also phagocytosis microglial debris, myelin, and synapses [7].

To better define the cellular source of phagocytic activity and its relationship to PNN remodeling in aging, we performed immunostaining for microglia (Iba1^+^), astrocytes (GFAP^+^), phagolysosomal activity (CD68^+^), and PNNs using *Wisteria floribunda* agglutinin (WFA^+^), enabling us to assess the spatial relationship between phagocytosis and PNN components.

## Materials & Methods

**Figure 1:**
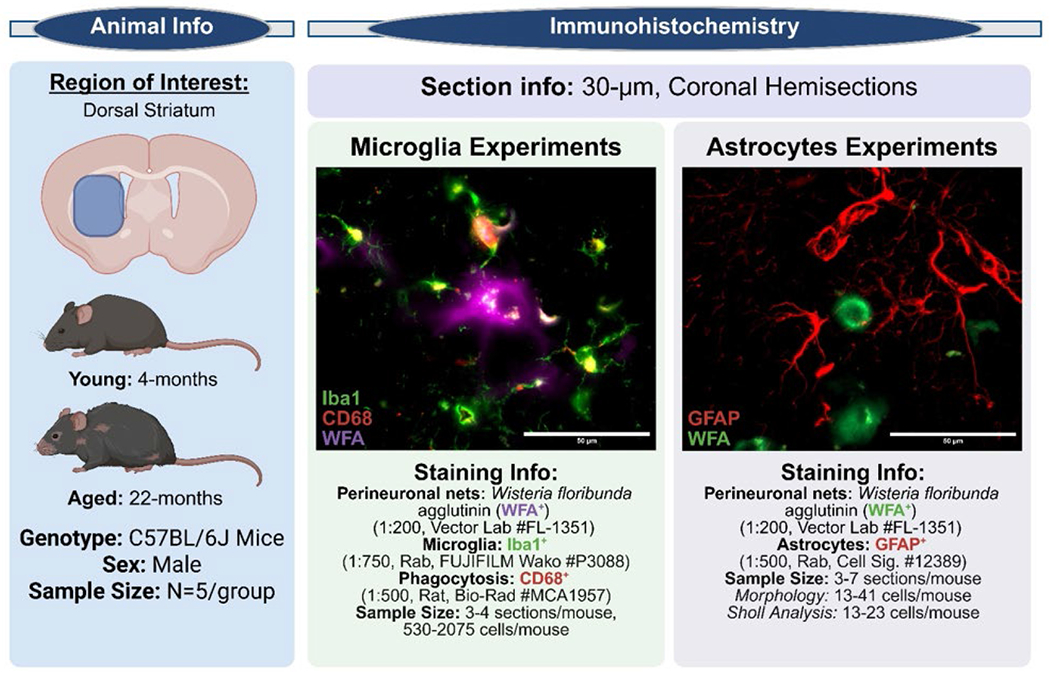
Experimental information.

Wild-type C57BL/6J mice (n = 5/group) were aged to 4-months (Young) or 22-months (Aged) and subjected to a battery of behavioral assessments prior to tissue collection for downstream RNA and immunohistochemical (IHC) analyses (previously published, Colon et al. 2025 [2]). Figure 1 outlines experimental details pertinent to this commentary

### Immunostaining

Brains were snap-frozen on dry ice, cryosectioned coronally at 30 μm on a sliding microtome (HM-400R, MICROM GmbH), and collected serially into a 12-well plate containing cryoprotectant for storage at −20°C. For each animal, one well containing 4–7 sections spanning the dorsal striatum (~300–360 µm apart) was processed for immunostaining. Free-floating sections were washed in 1X tris-buffered saline (TBS; 6 × 10 min), permeabilized in TBS with 0.1% Triton-X (TBST; 5 min), and blocked in TBST with 10% normal goat serum (NGS; 1 hr at room temperature). Sections were incubated overnight at 4°C with primary antibodies diluted in wash buffer (TBST with 1% NGS) with gentle shaking, followed by three washes (10 min each) and a 2-hr incubation with secondary antibodies at room temperature, protected from light. Sections were washed (3 × 10 min in wash buffer, 1 × 10 min in TBS), mounted onto slides, and coverslipped with Vectashield containing DAPI (Vector Laboratories, #H-1200). All primary and secondary antibodies were validated and optimized in-house prior to use.

Two independent staining cohorts were used. **Cohort 1:** microglia and phagocytic activity were assessed using anti-Iba1 (1:750, rabbit; FUJIFILM Wako, Cat# P3088) and anti-CD68 (1:500, rat; Bio-Rad, Cat# MCA1957), respectively, alongside fluorescein-conjugated *Wisteria floribunda* agglutinin (WFA; 1:200; Vector Laboratories, Cat# FL-1351) to label perineuronal nets. **Cohort 2:** astrocytes were labeled with anti-GFAP (1:500, rabbit; Cell Signaling Technology, Cat# 12389) together with WFA (1:200; Cat# FL-1351). Secondary antibodies included goat anti-rat Alexa Fluor 594 (1:1000; Invitrogen, Cat# A48264), goat anti-rabbit Alexa Fluor 635 (1:1000; Invitrogen, Cat# A31577), and goat anti-mouse Alexa Fluor 594 (1:1000; Invitrogen, Cat# A21125), applied as appropriate per cohort.

### Imaging

Researchers were blinded to conditions for imaging and analysis. Exposure times and laser intensities were optimized and maintained across samples. Imaging for cell counts were performed using a Zeiss Axio Imager.Z2 [13] microscope (20X/ air or 63X/oil objective). Images were stitched and processed using ZEISS ZEN (Blue Edition) [14] software (Carl Zeiss Microscopy) and FIJI (ImageJ) [15,16]. Tiled stitched images of the regions of interest with 30% overlap and stitched via Zen Blue “Stitching” function were used for 20X image analyses. Z-stacks of 9.84 μm, 42-slices, and 0.24 μm steps were used for 63X image analyses. Detailed microscope/imaging information can be found in Table 1.

### 20X Imaging Information

Raw 20X .czi images were stitched using EGFP as reference image with 30% minimal overlap and 32% maximal shift. Stitched images were opened on FIJI and channels were split. Images underwent background subtraction (rolling-ball radius = 50 pixels), Gaussian blur denoising (Sigma (radius) = 2), thresholded, despeckle filtering, and binarization followed by outlining of regions of interest (ROI) of the dorsal striatum with assistance of the Allen Mouse Brain Atlas [17,18] and added to ROI manager and outside of the ROI was cleared.

### Image processing for microglia experiments

The Cy5 channel used to image Iba1 (n=3-4 sections/mouse) underwent post-processing followed by the FIJI analyze particle function with the following parameters (Size (μm^2^) = 27-400, Circularity = 0.2-1.0). Cell counts were normalized to striatal area and ROIs were saved. The saved microglia ROIs (n = 530-2075 cells/mouse) were then used to measure intensity/microglia on raw stitched 20X .czi images for WFA^+^, CD68^+^, and Iba1^+^ intensity measures. PNNs were manually counted and saved to ROI manager.

### Image processing for astrocyte experiments

For cell counts, stitched tiled images (n = 3-7 sections/mouse) were manually counted using the AF594 (GFAP^+^) channel for each animal and normalized to striatal area. PNNs were manually counted and saved to ROI manager. For percentage of PNNs with a GFAP^+^ cells (Figure 3F), a PNN would be positive for association if one or more astrocyte is present within the PNN ROI. Data is presented as percentage of PNNs with an astrocyte present divided by total number of PNNs.

PNNs were randomly selected (n = 4/mouse) from anatomically matched sections spanning the rostrocaudal extent of the dorsal striatum, and all astrocytes within the imaging field of each selected PNN were included (n = 13-41 cells/mouse). Raw AF594 (GFAP^+^) z-stacks were acquired at 63X (9.58μm depth; 42 slices; 0.24μm steps). Images underwent background subtraction (rolling-ball radius = 100 pixels), despeckle filtering, thresholding, and binarization prior to maximum intensity z-projection. Cell body ROIs were manually outlined and saved to the ROI Manager. Astrocytes completely contained within the imaging field were used for endpoint and Sholl analysis (n = 13-23 cells/mouse). Sholl analysis was performed using the Neuroanatomy-Sholl Analysis FIJI plugin [19] with the following parameters: starting radius = 0 μm, ending radius = 100 μm, step size = 5 μm, 5-samples per radius, and full shells. Astrocyte distance to the nearest PNN was measured from the astrocytic soma to the closest PNN edge. For intensity measurements, saved ROIs were applied to average intensity z-projections of raw images to quantify mean GFAP and WFA fluorescence per cell.

### Linear regression analysis

The relationship between [Iba1^+^], [WFA^+^], and [CD68^+^] was assessed by Pearson’s correlation coefficient to examine the strength of association between the targets. Simple linear regression was performed using ordinary least squares fitting and statistical significance was determined by testing whether the slope of the regression differed from zero and if the slopes between groups were significantly different. For regression analyses, mean intensity of ROIs were observed and normalized to acquire an intensity value per animal for each variable. Normalized animal intensity values were plotted on x-y coordinates with variable [A] intensity along the x-axis and variable [B] intensities along the y-axis for each region of interest and each section. For each mouse, their intensity values were used to create an x-y coordinate and linear regression analysis was performed for each age group to calculate the coefficient of determination (R^2^) to identify significant relationships between the variables. Astrocyte [GFAP^+^] to [WFA^+^] linear regression analysis was performed with the same protocol.

## Results

### Normal aging increases the expression of CD68^+^ in microglia

Microglia within the aged dorsal striatum displayed canonical morphological features consistent with microgliosis, accompanied by elevated expression of *Cd68* (a lysosomal/phagocytic marker) and *Mmp9* (matrix metalloproteinase-9; a protease capable of degrading extracellular matrix and PNN components) [2]. Given that increased phagocytic capacity and proteolytic activity would be expected to compromise PNN integrity, the observation that overall PNN homeostasis was preserved suggests that these age-associated changes may not directly translate to enhanced PNN degradation, or alternatively that compensatory mechanisms maintain net stability despite heightened microglial activation [2].

**Figure 2.**
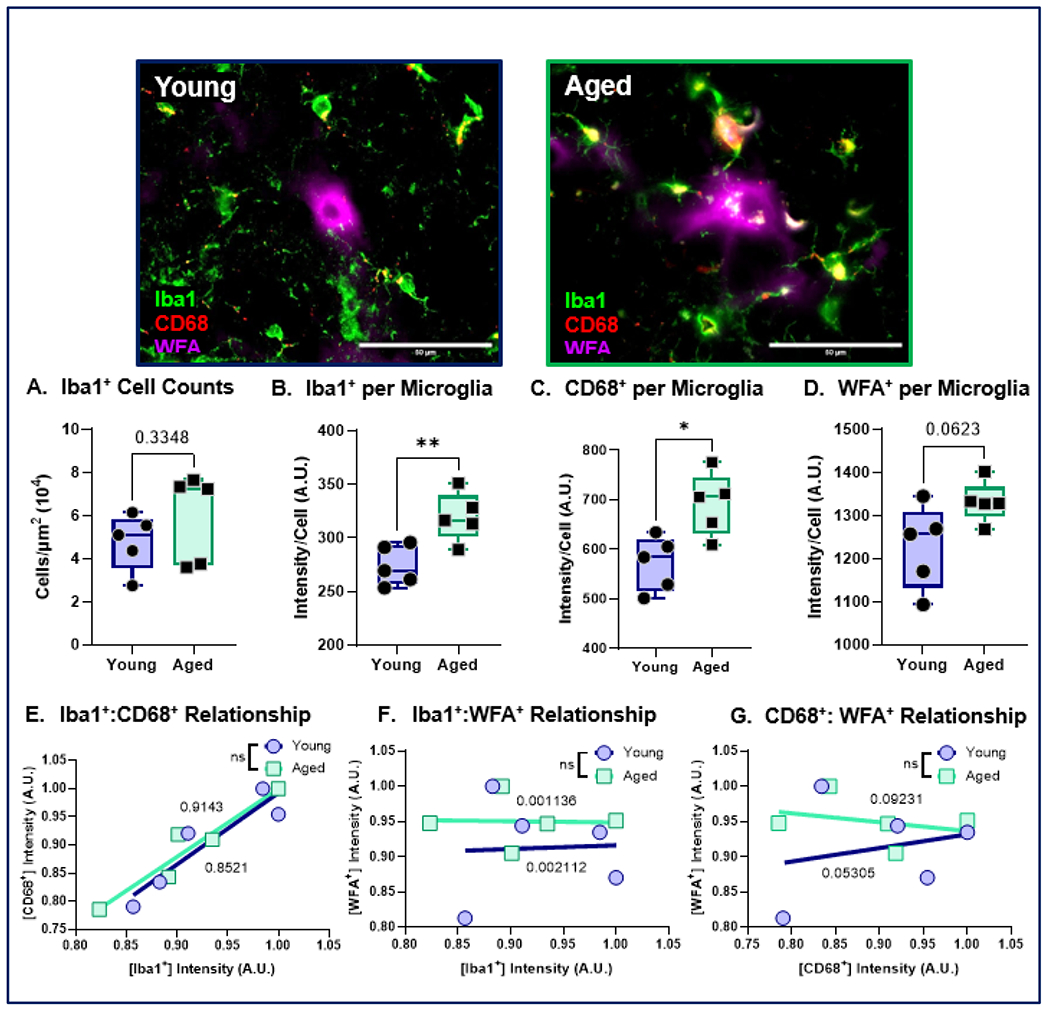
Normal aging promotes microglial activation and increased CD68^+^ expression in the dorsal striatum. **A.** Quantification of Iba1^+^ cell counts for the total striatum. Data was normalized to area. **B.** Quantification of Iba1^+^ intensity per Iba1^+^ cell. **C.** Quantification of CD68^+^ intensity per Iba1^+^ cell. **D.** Quantification of WFA^+^ intensity per Iba1^+^ cell. **E.** Linear regression analysis of Iba1^+^ intensity versus CD68^+^ intensity per cell. **F.** Linear regression analysis of Iba1^+^ intensity versus WFA^+^ intensity per cell. **G.** Linear regression analysis of CD68^+^ intensity versus WFA^+^ intensity per cell. Data represented as mean ± SEM, data was tested for normality prior to statistical analysis. Two-tailed t-tests or Welch’s t-test used when appropriate. Regression analysis: R^2^ values of corresponding linear regressions label on graph and t-test comparison of slopes used. p-values: <0.05, ** <0.01. N = 5 mice/group, 3–4 sections/mouse, 530–2075 cells/mouse.

To investigate microglia at the whole-region level, we detected no significant age-related differences in total microglial abundance (Figure 2A) within the dorsal striatum. However, single-cell analyses revealed significantly increased [Iba1^+^] and [CD68^+^] immunoreactivity per microglia in the Aged animals (Figures 2B and 2C), indicating an agedependent shift toward a more activated and lysosome-enriched microglial phenotype without gross changes in cell number. Additionally, aged mice exhibited an upward trend in [WFA^+^] signal per microglia (Figure 2D). Regression analyses demonstrated a significant positive relationship between [Iba1^+^] and [CD68^+^] expression in both Young and Aged animals (Figure 2E), supporting the interpretation that increased CD68 levels are coupled to microglial activation state. In contrast, no age-dependent association was observed between [Iba1^+^] and [WFA^+^] nor between [CD68^+^] and [WFA^+^] (Figures 2F–2G, respectively), suggesting that WFA^+^-labeled PNN-associated glycans are not robustly linked to microglial phago-lysosomal content under aging conditions. This raises the possibility that microglial engulfment may preferentially involve other non-glycan PNN constituents, warranting additional analyses using alternative PNN markers.

Collectively, these findings indicate that normal aging is associated with increased microglial activation and lysosomal/phagocytic marker expression at the single-cell level yet occurs without detectable disruption of overall PNN homeostasis. This dissociation supports the existence of compensatory regulatory processes—potentially including astrocyte-mediated maintenance of extracellular matrix structure—that preserve PNN integrity despite heightened microglial reactivity.

### Normal aging increases astrocyte proliferation and association with perineuronal nets, but astrocytes do not display reactive morphology

**Figure 3.**
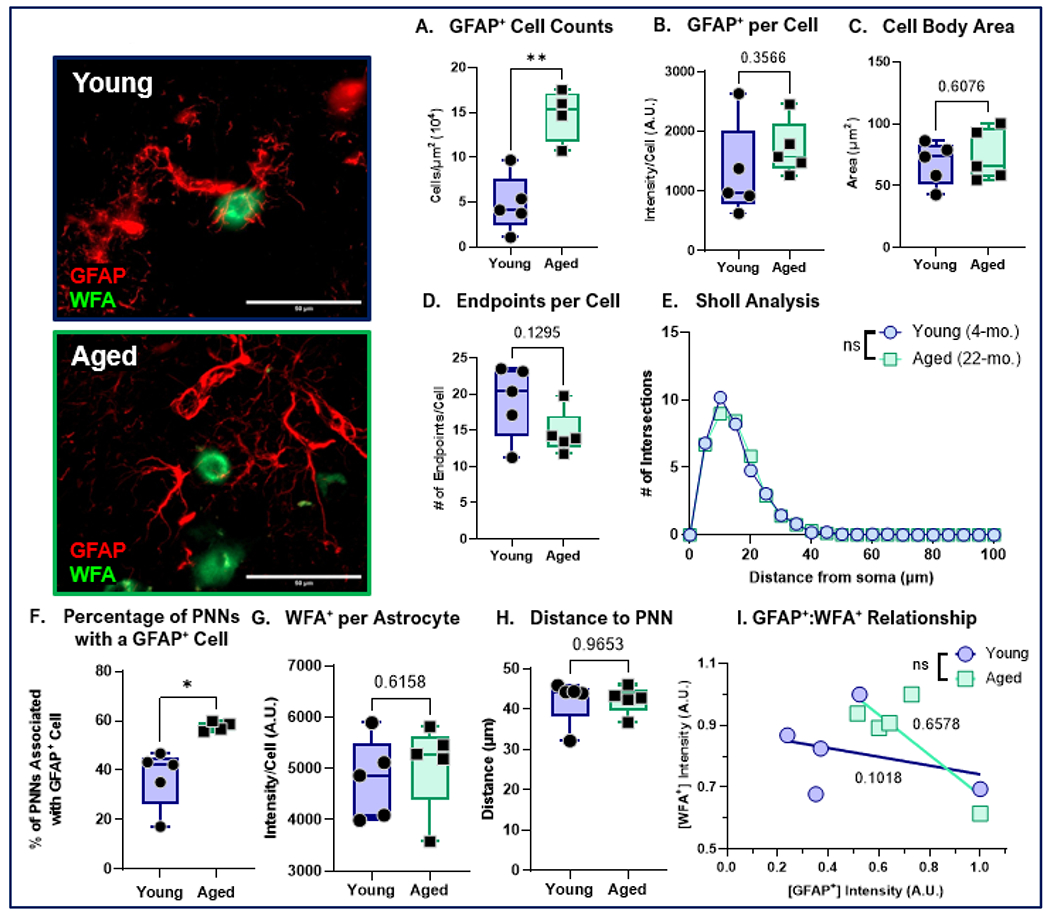
Normal aging increases astrocyte proliferation but does not increase reactive astrocytes. **A.** Quantification of GFAP^+^ cell counts for the total striatum. Data was normalized to area. **B.** Quantification of GFAP^+^ intensity per cell. **C.** Quantification of GFAP^+^ cell body area in the striatum. **D.** Quantification of average endpoints per GFAP^+^ cell in the striatum. **E.** Sholl analysis of the branch profile (ramification) of GFAP^+^ cells in the striatum. **F.** Percentage of PNNs associated with GFAP^+^ cells. **G.** Quantification of WFA^+^ intensity per GFAP^+^ cell. **H.** Quantification of distance of GFAP^+^ cells to soma of PNN-ensheathed neurons. **I.** Linear regression analysis of GFAP^+^ intensity versus WFA^+^ intensity per cell. R^2^ values of corresponding linear regressions label on graph. Data represented as mean ± SEM, data was tested for normality prior to statistical analysis. Two-tailed t-tests or Welch’s t-test used when appropriate. Sholl analysis: Two-Way ANOVA with Tukey. Regression analysis: t-test comparison of slopes. p-values: * <0.05, ** <0.01. N = 4–5 mice/group, 3–7 sections/mouse, Morphology: 13–41 cells/mouse, Sholl analysis/Endpoints: 13–23 cells/mouse.

To assess age-related changes in astrocytes, we next quantified astrocyte abundance, morphological features, and spatial interactions with perineuronal nets in the dorsal striatum. Quantitative RNA analyses revealed increased expression of *Gfap* and *C3*, two markers commonly associated with A1-like reactive astrocytes, raising the possibility that astrocytic reactivity increases during normal aging [2]. Consistent with this, aged mice exhibited a significant increase in total astrocyte number (Figure 3A). However, [GFAP^+^] intensity per astrocyte was not significantly altered (Figure 3B), suggesting that the elevated *Gfap* signal observed at the RNA level is more likely driven by increased astrocyte proliferation rather than enhanced GFAP^+^ expression per-cell.

To further evaluate astrocyte reactivity at the cellular level, we performed morphological analyses of astrocytes in proximity to perineuronal nets. Reactive astrocytes are typically characterized by hypertrophy, including increased soma size and altered process architecture (e.g., shorter, thicker ramifications) [20]. In our dataset, cell body area did not differ between the Young and Aged groups (Figure 3C). Similarly, measures of process complexity showed no age-related changes, including the number of endpoints per cell (Figure 3D) and process length/distribution (Figure 3E). Collectively, these findings support the interpretation that astrocytes increase in number during aging without exhibiting overt morphological hallmarks of reactivity, consistent with preservation of homeostatic astrocyte function in the aged striatum.

To define how astrocytes interact with PNNs during aging, we next quantified astrocyte–PNN spatial relationships. Regions of interest were generated around individual WFA^+^ PNNs throughout the dorsal striatum, and the proportion of PNNs closely associated with a GFAP^+^ astrocyte was calculated. Aged mice displayed a significantly higher percentage of PNNs associated with astrocytes (Figure 3F), indicating increased astrocytic coverage or recruitment to PNN-bearing neurons with age. This increased association was not accompanied by changes in WFA^+^ intensity within individual astrocytes (Figure 3G), astrocyte distance to the PNN-bearing neuronal soma (Figure 3H), or the degree of association of [GFAP^+^] to [WFA^+^] signal (Figure 3I). These data suggest that astrocytes are in close proximity to PNNs and that aging increases the number of astrocytes available to interact with these structures. Together, these findings support a model in which normal aging increases astrocyte abundance. This astrocytic expansion may serve as a compensatory mechanism that buffers the extracellular matrix against age-related increases in microglial activation, phagocytic signaling, and proteolytic pressure, thereby contributing to the maintenance of PNN homeostasis.

## Discussion

Perineuronal net homeostasis is a dynamic process that requires coordination between neurons, microglia, and astrocytes [11]. Microglia and astrocytes coordinate PNN regulation through bidirectional signaling that controls ECM production, remodeling, and degradation around PV interneurons [11]. Under homeostatic conditions, astrocytes support PNN maintenance by providing metabolic support and secreting key ECM components such as hyaluronan-associated chondroitin sulfate proteoglycans (CSPGs) and link proteins that stabilize net architecture [11,21]. In parallel, microglia remain in a surveillant phenotype and help preserve circuit integrity through trophic support and limited remodeling. During aging, microglial activation increases the release of cytokines and chemokines (e.g., IL-6, TNF-α) [22], which would be predicted to promote astrocyte reactivity and shifts astrocytic output toward altered CSPG deposition and increased expression of ECM-modifying proteases. Here we report that despite increases inflammation as well as microglial activation and phagocytosis there is no change in the number of PNNs. While CD68^+^ is increased in the aged microglia and we show a trend towards increased WFA intensity/microglia, the possibility remains that microglia are also targeting synapses, myelin debris, and protein aggregates for phagocytosis. Alternatively, the increased CD68/microglia could reflect an intracellular lysosomal buildup within microglia.

The exact function of aged reactive microglia and the coordinated glial communication that acts to balance PNN stabilization and degradation/remodeling remains unclear and warrants further investigation. It is possible that the proliferation of astrocytes in aged mice is a compensatory mechanism working to balance increased microgliosis. Prior work has demonstrated that experimental removal of PNNs can promote compensatory expansion of astrocytic pericellular coverage around PV interneurons without significantly altering synaptic transmission, suggesting that astrocytes may dynamically adapt to changes in the perineuronal extracellular matrix [23]. In addition, PV interneurons have exceptionally high metabolic demands, and astrocytes are essential for sustaining their activity through metabolic coupling mechanisms such as the astrocyte–neuron lactate shuttle [24,25]. A caveat is that while the use of GFAP is well established measure for astrogliosis, it is a cytoskeletal protein leaving detailed measures on the astrocytic coverage on the soma of PNN-containing cells remains limited.

## Conclusion

Overall, these findings support an age-related shift toward microglia activation without producing overt disruption of PNN homeostasis. At the single-cell level, microglia in Aged mice exhibited increased Iba1^+^ and CD68^+^ expression, consistent with a more activated, lysosome-enriched phenotype. In parallel, aging increased astrocyte abundance and astrocyte–PNN association without clear morphological hallmarks of reactivity, raising the possibility that astrocytes provide a compensatory stabilizing influence that buffers PNN structure against increased microglial inflammatory and proteolytic pressure. Together, these data suggest that PNNs may remain stable under baseline conditions but potentially vulnerable to degradation following a secondary inflammatory insult, highlighting the importance of coordinated glial communication in maintaining extracellular matrix integrity. We are actively continuing to investigate how microglia and astrocytes interact with PNNs across aging and inflammatory conditions, so stay tuned.

## Figures and Tables

**Table 1.** Microscope and imaging information.

Experiment	Microglia Experiments	Astrocyte Experiments	
** Figure Number**	**Figure 2**	**Figure 3A/3F**	**Figure 3B–E/3G–I**
**Microscope**	Zeiss AxioImager.Z2	Zeiss AxioImager.Z2	Zeiss AxioImager.Z2
**Objective**	Plan-Apochromat 20×/0.8 M27	Plan-Apochromat 20×/0.8 M27	Plan-Apochromat 63×/1.4 Oil M27 M27
**Scaling (per Pixel)**	0.172um × 0.172um	0.172um × 0.172um	0.055um × 0.055um × 0.240um
**Bit Depth**	14-bit	14-bit	14-bit
**Effective NA**	0.8	0.8	0.8
** Imaging Device**	705m-Fluou	705m-Fluou	705m-Fluou
**Tiling Information**	30% Overlap, Minimal Overlap = 30%, Maximal Shift = 32%	30% Overlap, Minimal Overlap = 30%, Maximal Shift = 32%	NA
**Tiling Reference Channel**	EGFP	EGFP	
**Z-stack (Stack size) (μm)**	NA	NA	9.84
**Z-stack (Step size) (μm)**			0.24
**Z-stack (number of slices)**			42
** Channel/Target**	**DAPI/Nuclei**	**DAPI/Nuclei**	**DAPI/Nuclei**
** Excitation (nm)**	353	353	353
**Emission (nm)**	465	465	465
**Intensity**	25%	25%	25%
**Exposure**	26	20	50
** Depth of Focus**	1.45	1.45	0.72
** Channel/Target**	**EGFP/WFA**	**EGFP/WFA**	**EGFP/WFA**
** Excitation (nm)**	488	488	488
**Emission (nm)**	509	509	509
**Intensity**	25%	10%	25%
**Exposure**	80	90	100
** Depth of Focus**	1.59	1.59	0.79
** Channel/Target**	**AF594/CD68**	**AF594/GFAP**	**AF594/GFAP**
** Excitation (nm)**	590	590	590
**Emission (nm)**	618	618	618
**Intensity**	25%	20%	20%
**Exposure**	50	110	135.3
** Depth of Focus**	1.93	1.93	0.96
** Channel/Target**	**Cy5/Iba1**	NA	NA
** Excitation (nm)**	650		
**Emission (nm)**	673		
**Intensity**	25%		
**Exposure**	200		
** Depth of Focus**	2.1		
**Postprocessing Information**			
**Background Subtraction (pixels)**	50	50	100
**Gaussian Blur (sigma)**	2	2	2
**Threshold**	Variable	Variable	Variable
**Analyze Particles Setting**	Size (um^2^) = 27–400, Circularity 0.2–1.0	NA	NA
**Z-projection**	NA	NA	Morphology: “Max Intensity”, Intensity: “Average Intensity”
